# Simultaneous onset of familial hemophagocytic lymphohistiocytosis and T-cell large granular lymphocytic leukemia in an adult

**DOI:** 10.1007/s00277-026-07177-9

**Published:** 2026-07-15

**Authors:** Friederike Dierks, Finn-Ole Paulsen, Kai Lehmberg, Andreas Rosenwald, Carsten Bokemeyer, Katja Weisel, Christoph Seidel

**Affiliations:** 1 Department of Oncology, Hematology and Bone Marrow Transplantation with Division of Pneumology, University Medical Center Hamburg-Eppendorf, Hamburg, Germany; 2 Division of Pediatric Stem Cell Transplantation and Immunology, Department of Pediatric Hematology and Oncology, University Medical Center Hamburg-Eppendorf, Hamburg, Germany; 3 Institute of Pathology, University of Würzburg, Würzburg, Germany

**Keywords:** Hemophagocytic lymphohistiocytosis, Adult onset, T-cell large granular lymphocytic leukemia, Case report

## Abstract

Hemophagocytic lymphohistiocytosis (HLH) is a severe hyperinflammatory syndrome. The familial form (fHL), associated with specific genetic alterations, typically manifests in childhood, while secondary forms arise due to triggers such as malignancies or infections. We present the rare case of adult-onset fHL in a 30-year-old female concurrent with T-cell large granular lymphocytic leukemia (T-LGL). After presenting with symptoms of HLH, a bone marrow biopsy revealed T-cell lymphoma, leading to CHOEP treatment. This diagnosis was later revised to T-LGL, prompting a switch to methotrexate for T-LGL treatment. Further genetic testing uncovered an unexpected homozygous missense mutation in *UNC13D* (p.R414C), associated with fHL type 3. A detailed family history revealed a consanguineous marriage between her parents, which likely increased the risk of the homozygous mutation. Additionally, it was discovered that the patient’s sister had undergone allogeneic stem cell transplantation during childhood for an unspecified lymphoproliferative disorder, with the patient herself serving as the donor. This case highlights the rare occurrence of adult-onset fHL, triggered by the evolution of T-LGL as a possible contributing factor. It underscores the importance of early diagnosis and illustrates how familial history and persistent symptoms can necessitate genetic testing.

## Introduction

Hemophagocytic lymphohistiocytosis (HLH) is a severe syndrome caused by hyperinflammation due to abnormal immune activation. It includes a familial form (fHL), which is associated with specific genetic alterations and typically manifests in early childhood, and secondary forms, triggered by various conditions such as infections or lymphomas [1]. Subtypes of fHL are classified based on mutations affecting cytolytic vesicle function or perforin function [2]. Type 2 involves mutations in *PRF1* on chromosome 10q21, which impair the cytolytic activity of immune cells, such as T cells, by reducing perforin [3–5]. fHL types 3–5 are associated with defective cytotoxic granule exocytosis [6], with mutations occurring in *UNC13D* on chromosome 17p25 in type 3 [7], *STX11* on chromosome 6q24 in type 4 [8], and *STXBP2* on chromosome 19p13 in type 5 [9, 10].

The various deficiencies in fHL can facilitate rapid testing alongside molecular diagnostics to distinguish familial from secondary forms. For example, detection of perforin or Granzyme B can aid in differentiation [9]. Flow cytometry for CD107a, a marker of lytic granule exocytosis, can confirm types 3–5, as 88% of cases showed abnormal NK-cell degranulation in studies [11]. Additionally, stimulation with IL-2 can help distinguish phenotypes, as it enhances degranulation, particularly in fHL types 4 and 5 [11].

The subtypes of HLH share characteristic symptoms, first defined in the HLH-94 study and later revised in the HLH-2004 study. A diagnosis is confirmed either by molecular diagnosis or if five of the following eight symptoms are present: fever, splenomegaly, bicytopenia, hypertriglyceridemia, hemophagocytosis, hyperferritinemia, low NK cell activity, or elevated soluble IL-2 receptor-α (sCD25) [1]. Revised guidelines include functional cellular findings in patients with suggestive symptoms as criteria for diagnosis of fHL [12]. Alternatively, the “H-Score” can be used to calculate the likelihood of HLH by assessing nine variables. A score above 169 indicates a sensitivity of 93% and specificity of 86% for HLH [13].

In cases of fHL, treatment follows the HLH-94 protocol, which includes dexamethasone, etoposide, and cyclosporine A, with methotrexate added if there is neurological involvement. Hematopoietic stem cell transplantation (SCT) remains the only long-term curative option [14, 15].

## Case presentation

**Fig. 1:**
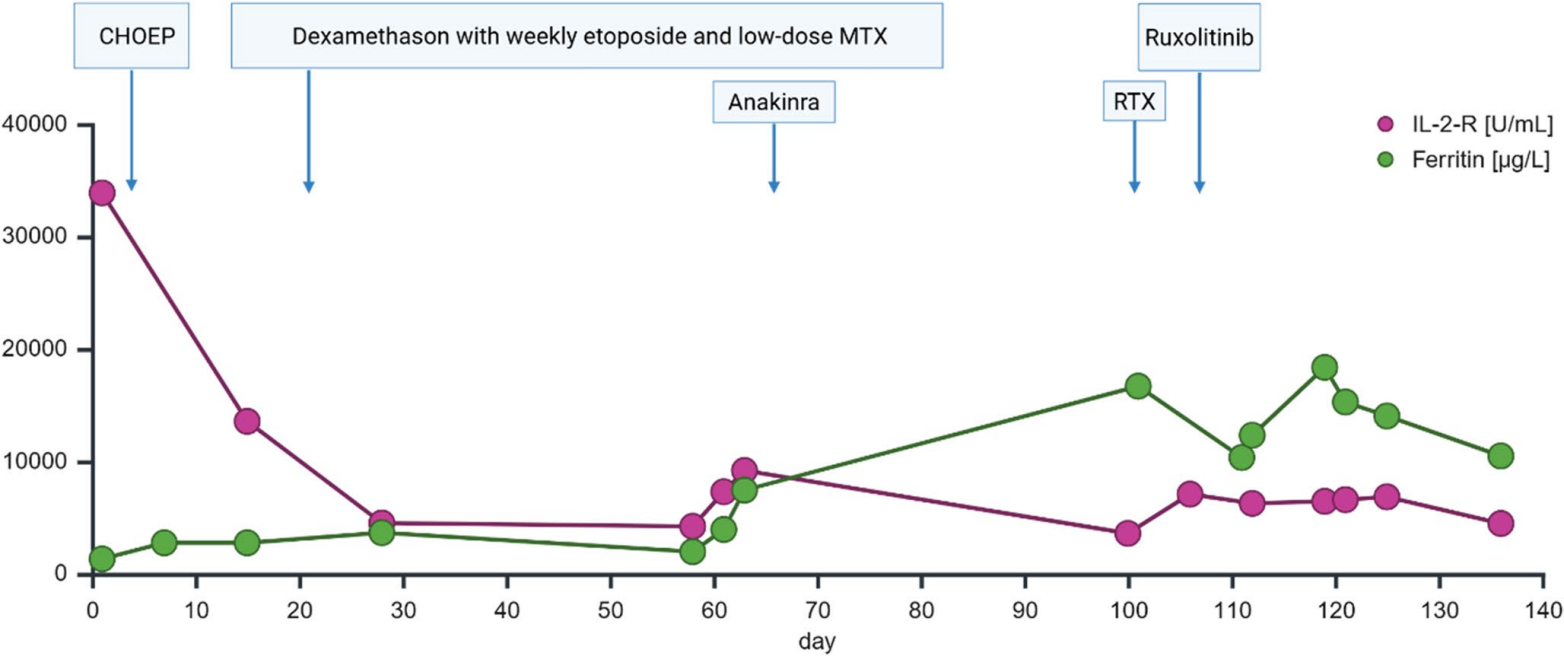
Levels of ferritin and IL-2-receptor during treatment

We present the case of a 30-year-old female who was referred to our center with persistent fever and splenomegaly. Initial laboratory testing revealed pancytopenia (Hb 7.8 g/dL (extern: 5,9 g/dL), leukocytes 3.2 × 10⁹/L, platelets 23 × 10⁹/L), hyperferritinemia (1108.5 µg/L), hypertriglyceridemia (301 mg/dL), elevated ASAT (46 U/L), and elevated sCD25 (33,876 U/mL), consistent with a diagnosis of HLH (initial H-Score: 218 points). Further diagnostic work-up included bone marrow biopsy, liver biopsy, and PET-CT scan. The PET-CT scan revealed increased metabolic activity in the spleen and bone marrow, while pathological assessments of the liver and bone marrow biopsy confirmed the presence of T-cell lymphoma with increased macrophage infiltration. This prompted emergency chemotherapy with the CHOEP regimen (cyclophosphamide, doxorubicin, vincristine, etoposide, and prednisolone) to address the suspected high-grade T-cell lymphoma. Consistent with the suspected secondary HLH, the treatment led to immediate stabilization of symptoms, supported by a decrease in soluble IL-2 receptor levels and a consistent reduction in ferritin values, both of which can be used to monitor active HLH (Fig. 1).

The timeline presents the changes of IL-2-receptor [U/mL] and ferritin [µg/L] during treatment over the course of 136 days. Initially emergency treatment with CHOEP was initiated with adjustment to dexamethasone with weekly etoposide according to the HLH-94-protocol and low-dose MTX addressin T-cell large granular lymphocytice leukemia (T-LGL). Although decrease of T-LGL-cells was observed, no clinical improvement was shown, leading to subsequent treatment with Anakinra and Ruxolitinib. RTX (Rituximab).

Contrary to the initial diagnosis, further specified hematopathological analysis identified the monoclonal T-cell population previously described to be compatible with a low-grade T-cell malignancy in form of T-LGL with immunophenotypically detection of CD3+, CD8+, Perforin/ Granzym B/ TIA1+, CD57+, CD56- cells. The presence of T-LGL cells were also confirmed in the peripheral blood by detection of azurophilic granules and 13,65% CD3 + cells with co-expression of CD57. No mutations in STAT3 or STAT5B were identified. LGL describes a lymphoproliferative disorder evolving from either T- or NK-cells. It is defined by an elevated clonal expansion due to activation of survival pathways like Jak/Stat. In our case, after confirmed diagnosis of T-LGL, treatment was adjusted according to the HLH-94-protocol with dexamethasone and weekly etoposide. Furthermore the patients received low-dose MTX addressing LGL [2, 14, 16] (Fig. 1).

Despite a reduced T-LGL cell count and diminished signs of HLH in the bone marrow in subsequent observations, clinical improvement remained incomplete. To investigate other potential causes of HLH, flow cytometry was performed, and confirmed normal perforin expression but identified a degranulation deficiency through CD107a detection. Subsequent genetic testing revealed a homozygous missense mutation in *UNC13D* (p.R414C), associated with fHL type 3 [7].

In line with the diagnosis of fHL, a detailed family history revealed a consanguineous marriage of both her parents and herself. Additionally, family history disclosed that the patient’s sister had undergone allogeneic stem cell transplantation during childhood for a lymphoproliferative disorder, with the patient serving as the donor. The current condition of sister and the patient’s child remain unknown.

Regarding further treatment, SCT was proposed as the only curative option [1]. To prevent potential aggravation of HLH due to concurrently detected CMV and EBV viremia, the patient was administered Ganciclovir and Rituximab (RTX). Given the patient’s unstable condition, the therapy was further adjusted to include the interleukin-1 receptor antagonist Anakinra and, subsequently, the JAK 1/2 inhibitor Ruxolitinib. Despite these adjustments, the treatment remained insufficient. Unfortunately, the patient succumbed to fatal mucormycosis and infectious complications before undergoing SCT.

## Discussion

This case highlights the rare occurrence of adult-onset familial fHL unmasked by the evolution of T-LGL as a potential trigger factor. It underscores the importance of obtaining a detailed family history, as it revealed the consanguineous marriage of the patient’s parents, which increases the likelihood of homozygous mutations [17]. The presence of a sibling with a severe, unresolved illness might also be indicative [18].

While fHL often occurs during the first year of life, cases occurring in adulthood, partly also with homozygous *UNC13D* mutations, have been documented in the literature [19, 20]. A potential cause for adult-onset fHL could be a higher prevalence of missense mutations that impair protein function upon provocation, whereas in pediatric patients, nonsense or frame-shift mutations that abolish protein function are more common [19, 21].

Considering the overrepresentation of *UNC13D* haploinsufficiency in patients with lymphoma [22], particularly leukemia [17], it is crucial to understand the underlying association between fHL type 3 and cancer. Hematological malignancies are likely more frequent in patients with hypomorphic cytotoxicity defects due to reduced immune surveillance [22, 23]. The observed T-LGL in this case may be associated with a genetic predisposition, as T-LGL predominantly affects elderly individuals, with only 25% occurring in those younger than 50 years [16]. However, the correlation between the rare occurrence of T-LGL in this young patient and the *UNC13D* mutation causing fHL type 3 remains unclear and warrants further investigation.

The possible transfer of the OHI index, which was developed for malignancy-associated HLH and facilitates diagnosis by assessing sCD25 and ferritin levels and is predictive of mortality, could also be of interest in case of fHL [24].

Overall, this case offers new insights into the possible circumstances surrounding late-onset fHL and highlights the importance of considering HLH early in the differential diagnosis, not only in children but also in adults. Detailed familial anamnesis, especially in patients with persistent symptoms and inadequate response to seemingly adequate treatment for secondary HLH, is crucial for early genetic testing. Early treatment based on clinical diagnosis, even before genetic confirmation, may significantly impact outcomes in cases of relentlessly progressing disease [9, 14, 15].

## Data Availability

No datasets were generated or analysed during the current study.

## Abbreviations

fHL: Familial HLH
HLH: Hemophagocytic lymphohistiocytosis
LGL: T-cell large granular lymphocyte leukemia
SCT: Hematopoietic stem cell transplantation
RTX: Rituximab
